# Dendritic Cells Take up and Present Antigens from Viable and Apoptotic Polymorphonuclear Leukocytes

**DOI:** 10.1371/journal.pone.0029300

**Published:** 2011-12-20

**Authors:** Carlos Alfaro, Natalia Suarez, Carmen Oñate, Jose L. Perez-Gracia, Ivan Martinez-Forero, Sandra Hervas-Stubbs, Inmaculada Rodriguez, Guiomar Perez, Elixabet Bolaños, Asis Palazon, Miguel Fernandez de Sanmamed, Aizea Morales-Kastresana, Alvaro Gonzalez, Ignacio Melero

**Affiliations:** 1 Gene Therapy Unit, CIMA, Universidad de Navarra, Pamplona, Spain; 2 Medical Oncology Department, Clínica Universidad de Navarra, Universidad de Navarra, Pamplona, Spain; 3 Biochemistry Department, Clínica Universidad de Navarra, Universidad de Navarra, Pamplona, Spain; Singapore Institute for Clinical Sciences, Singapore

## Abstract

Dendritic cells (DC) are endowed with the ability to cross-present antigens from other cell types to cognate T cells. DC are poised to meet polymorphonuclear leukocytes (PMNs) as a result of being co-attracted by interleukin-8 (IL-8), for instance as produced by tumor cells or infected tissue. Human monocyte-derived and mouse bone marrow-derived DC can readily internalize viable or UV-irradiated PMNs. Such internalization was abrogated at 4°C and partly inhibited by anti-CD18 mAb. In mice, DC which had internalized PMNs containing electroporated ovalbumin (OVA) protein, were able to cross-present the antigen to CD8 (OT-1) and CD4 (OT-2) TCR-transgenic T cells. Moreover, in humans, tumor cell debris is internalized by PMNs and the tumor-cell material can be subsequently taken up from the immunomagnetically re-isolated PMNs by DC. Importantly, if human neutrophils had endocytosed bacteria, they were able to trigger the maturation program of the DC. Moreover, when mouse PMNs with *E. coli* in their interior are co-injected in the foot pad with DC, many DC loaded with fluorescent material from the PMNs reach draining lymph nodes. Using CT26 (H-2^d^) mouse tumor cells, it was observed that if tumor cells are intracellularly loaded with OVA protein and UV-irradiated, they become phagocytic prey of H-2^d^ PMNs. If such PMNs, that cannot present antigens to OT-1 T cells, are immunomagnetically re-isolated and phagocytosed by H-2^b^ DC, such DC productively cross-present OVA antigen determinants to OT-1 T cells. Cross-presentation to adoptively transferred OT-1 lymphocytes at draining lymph nodes also take place when OVA-loaded PMNs (H-2^d^) are coinjected in the footpad of mice with autologous DC (H-2^b^). In summary, our results indicate that antigens phagocytosed by short-lived PMNs can be in turn internalized and productively cross-presented by DC.

## Introduction

Neutrophils are the first defense barrier against bacteria or fungi that have penetrated the body surfaces. Many features equip these cells to perform such a critical function [1]. Originating in the bone marrow, neutrophils have a short 4 h half life in circulation and readily extravasate upon infection or inflammation in any infected tissue [2], [3]. Neutrophils sense and migrate in response to chemotactic gradients at inflammatory sites [2]. Interleukin-8 [4], [5], bacterial formyl peptides and complement factors are the main chemotactic stimuli, while extravasation is guided by LFA-1 integrin interactions with ICAM-1 [2], [6]. Once in contact with the invading microorganisms, neutrophils degranulate microbiocide substances, perform phagocytosis and oxidative killing of microorganisms [1], [7], take up tissular debris, and entrap bacteria by means of forming trapping nets with their own DNA [1], [8]. Pus as it appears in acute inflamed foci is a collection of exudates rich in the remains of neutrophils, microorganisms and tissular debris.

Adaptive immune responses rely on antigen presentation to T-cells as performed by professional dendritic cells (DC) [9]. At least some of the DC subsets present in the body can take-up antigen from third party cells and present them to lymphocytes associated to MHC class I and II molecules [10]. In the case of MHC class I-mediated presentation, this process is referred to as antigen cross-presentation [11]. There is recent evidence indicating that various human DC subsets isolated from human can mediate cross-presentation [12]. Indeed, the CD141^+^ CLEC-9A^+^ subset seems to be specialized in CD8 T-cell cross-priming [13], .

The ability of DC to stimulate a T-cell response is not constitutive [16]. On the contrary, DC depend on detection of microbial molecular patterns [17], tissue damage denoting molecules [18] and proinflamatory cytokines to experience their maturation program. Maturation means migration towards lymphoid organs triggered by CCR7 ligands [19], upregulation of co-stimulatory molecules for T cells and production of T-cell stimulating cytokines [17].

An interesting article indicated that DC and neutrophils may interact in the body in sites of acute inflammation such as a phlegmonous appendicitis [20]. Importantly the Van Kooyk group demonstrated that a molecular interaction dependent on DC-SIGN on the DC side and sialyl Lewis-X sugars anchored on the CD11b integrin mediated the heterotypic adhesion of human PMN to DC [20], [21]. Other authors have shown that neutrophils enhance the expression of co-stimulatory molecules on DC [22], [23]. There have been also reports of the ability of neutrophils to directly present antigen to T cells [24].

From our point of view, it makes physiological and economic sense that scanty DC would be able to interact with abundant neutrophils that bear signs of having internalized relevant microbiological antigens. Such stressed PMN leukocytes would be thereby a concentrated source of microbiological antigens. Even under sterile conditions neutrophil granule proteins have been observed to elicit maturation of DC [23], [25], [26]. However, it is clear that if DC internalized infected neutrophils this would not only constitute a rich source of microbial antigens, but also a concentrated source of microbial molecular patterns to elicit DC maturation. If this hypothesis is correct, the ability of PMNs to ferry antigenic material for DC efficient cross-presentation might be useful in cancer immunotherapy.

We found that human and mouse DC internalize viable and dead PMNs. Interestingly, if such PMN had been pre-exposed to dying tumor cells, the tumor-related material ends up being taken up by DC. We demonstrate in mice that antigens internalized by PMNs can be cross-presented by DC. Furthermore, intracellular antigens from dead tumor cells can be ferried by PMNs to be readily up-taken by DC and subsequently cross-presented to CD8^+^ T cells. The importance of second and third hand antigen presentation might have been overlooked, and PMN leukocytes may play a critical role as a source of concentrated antigen and in providing maturation stimuli to DC.

## Materials and Methods

### Ethics statement

Animal studies have been performed in accordance with Spanish legislation under specific approval from the institutional ethics board by the *Ethics Committee for Animal Experimentation of the University of Navarra* (Study 03/007 approval). Human cells are obtained from Blood donors (public blood bank of Navarra) under written informed consent for research.

### Purification of human neutrophils

10 ml of peripheral blood drawn in heparin containing tubes were mixed with 10 ml of cold PBS and 10 ml of 6% Dextran/0.9% NaCl solution. After being inverted 18–20 times to ensure adequate mixing, the mixture was pipetted into a 50 ml tube and the tube was placed upright at room temperature for 1–1.5 h, or until separation was completed. The yellowish supernatant was recovered into a 50 ml tube and spun at 1150 rpm for 12 minutes at 4°C with low brake. The supernatant was then discarded and the pellet re-suspended in 5 ml ACK buffer, 5 min at room temperature. After that incubation, the cells were washed with 45 ml of cold PBS. The supernatant was discarded and the pellet was resuspended in 2.5 ml of PBS. The cell suspension was laid over 3 ml of Ficoll-Hypaque (GE Healthcare Europe GMBH, Barcelona, Spain) gradients in a 15 ml tube and spun at 1500 rpm for 30 minutes at 4°C. The pellet was resuspended in 2 ml cold PBS and stored at 4°C. To ensure the correct purification the pellet was immunostained with CD15-PE and a Giemsa staining of cytospins was performed. Neutrophil purity was >95% (CD15^bright^ neutrophils) [27].

### Purification of mouse neutrophils

BALB/c mice neutrophils were obtained from bone marrow as follows. Hind limbs were collected in ethanol and bones were cleaned with a scalpel (peeling off as much flesh as possible). When all bones were cleaned, their ends were cut and a 27G1/2 needle placed in the central cavity of the long bones to flush them with mouse complete medium [RPMI 10% FBS (Invitrogen, Paisley, UK) + penicillin/streptomycin (Invitrogen) + β-mercaptoethanol (Invitrogen)]. An immunomagnetic selection was made with Ly6G AutoMACS microbeads (Miltenyi Biotec, Bergisch Gladbach, Germany) obtaining the positive and the negative fractions to monitor cell population enrichment. Anti-Gr-1-biotin (Becton Dickinson, Erembodegem, Belgium) plus streptavidin-PE (BD Bioscience) was used for monitoring enrichment by flow cytometry using a FACSCalibur Flow Cytometer (Becton Dickinson). It is of note that the anti-Gr-1 mAb recognizes both Ly-6C and Ly-6G.

### Human DC generation and maturation

DC were generated from filter buffy coat (FBC)-derived monocytes donated by healthy human donors [28]. The protocol for obtaining FBC was approved by the Ethics Committee of our institution and donors gave informed consent. Isolated mononuclear cells were subjected to positive selection using anti-CD14-conjugated paramagnetic beads and purified using the AutoMACS system according to manufactureŕs instructions (Miltenyi Biotec). Purified monocytes were cultured for 7 days in complete medium (RPMI 10% FBS + penicillin/streptomycin + β-mercaptoethanol) supplemented with GM-CSF (1000 U/ml; Leukine, Schering-Plough, Kenilworth, NJ, USA) and IL-4 (500 U/ml; R&D Systems, Minneapolis, MN, USA). Cytokines were added every other day. DC were matured with clinical grade TNFα (50 ng/ml; Boehringer Ingelheim, Ingelheim, Germany), IFNα (1,000 IU/ml; Schering-Plough) and Poly I:C (20 µg/ml; Ampligen, Bioclones, South Africa) during 48 h [29].

### Murine DC generation

Mice DC precursors were obtained from bone marrow. Cells were put in culture [RPMI 10% FBS (Invitrogen) + penicillin/streptomycin (Invitrogen) + β-mercaptoethanol (Invitrogen)] at a density of 2×10^6^ cells/ml in 10 ml per petri dish in the presence of 20 ng/ml of rmGM-CSF (R&D) [30]. After 72 h, rmGM-CSF at 10 ng/ml was added in 10 ml in RPMI complete medium per dish. On 6th day of culture, 10 ml of medium were withdrawn per dish. On 9th culture day, non adherent cells were collected by thoroughly pipetting with RPMI medium. Adherent cells were collected by adding 2 ml of accutase (PAA laboratories GmbH, Pasching, Austria) per dish and incubated 15 minutes at 37°C.

### Interaction between DC and neutrophils *in vitro*

10^6^ DC were stained with PKH26 and 5×10^6^ neutrophils with PKH2 membrane marker (Sigma-Aldrich, St. Louis, MO, USA) as reported [27]. Half of the neutrophils were killed with UV light at 260 nm (1,232 J/cm^2^). After that, live or dead neutrophils were co-cultured with DC for 4 and 24 hours at 4°C or 37°C. The neutralizing antibodies anti-CD11b, anti-CD18 (kindly provided by Francisco Sanchez Madrid, Hospital de la Princesa, Madrid) and anti-DC-SIGN (kindly provided by Angel Corbí, CIB, Madrid) were added to the culture medium at 10 µg/ml or at a 1/5 dilution of the concentrated hybridoma supernatant. After 2, 4 and 24 hours, co-culture cells were collected to analyse double positive cells using a FACSCalibur Flow Cytometer or examined by confocal microscopy.

### *In vitro* chemotaxis assay

*In vitro* neutrophil and DC migration was measured in Transwell Chambers (5 µm; Corning Costar, Corning, NY, USA). Both PKH26-DCs (10^5^) and PKH2-labeled neutrophils (10^5^) were added to the upper chamber and migration stimuli (subconfluent monolayer of HT29 cells) were placed in the lower chamber. DC and neutrophils were added to the upper chamber in complete medium, complete medium plus 20 µg/ml antiIL-8 mAb (BD Pharmigen), or complete medium plus mIgG2b as control. Transmigrated cells in the lower chamber were quantified using fluorescence microscopy imaging of the lower chamber. The number of PKH26-fluorescent DC or PKH2-labeled neutrophils per microscopic field (x20) in the lower chamber was quantitated in triplicate wells by a blinded observer.

### Flow cytometry analyses

FITC and PE-labeled mAb specific for the DC maturation markers CD80, CD83, CD86 and HLA-DR (BD Bioscience) and isotype-matched labeled controls were used to characterize cell surface phenotypes by flow cytometry. PE-labeled mAb specific for CD15 and Gr-1-biotin + SAV-PE (BD Bioscience) were used to analyse purity in human and mice leukocyte samples. Splenocytes were obtained from OT-1 and OT-2 TCR transgenic mice (Jackson Laboratories, Bar Harbor, ME, USA) [31]. Lymphocyte division was analysed by serial dilution of carboxifluorescein succinimidyl ester (CFSE) in CD4^+^ and CD8^+^ cells (BD Bioscience). Cells (10^5^) were washed in cold PBS and incubated 15 min at 4°C with specific APC, FITC or PE-labeled Abs to electronically gate CD4 and CD8 cells upon FACS analyses.

### Immunofluorescence using confocal microscopy

Confocal fluorescent images were obtained using a LSM 510 Zeiss confocal scan head mounted on a Zeiss Axiovert 200 M on an inverted-based microscope using a 40x or 63x oil immersion objective. Sequential excitation at 488 nm and 543 nm was provided by argon and helium-neon gas lasers, respectively. Emission filters BP500-550 and LP560 were used for collecting green (PKH2) and red (PKH26) in channels one and two, respectively. After sequential excitation, green and red fluorescent images of the same cell were saved with Laser Sharp software. Images were analyzed by Zeiss software. The term co-localization refers to the coincidence of green and red fluorescence, as measured by the confocal microscope.

### Protein quantification

After culturing the neutrophils in complete medium supplemented with 1 mg/ml of OVA protein (Sigma-Aldrich) for 2 h, the supernatants of serial centrifugations were collected to verify the complete elimination of extracellular OVA. Protein concentration was measured in a Nanodrop Spectrometer ND-1000 at 280 nm. Stock solution was used as control.

### Chemokine production

Supernatants were collected in the indicated culture time points, and the cytokine concentration was determined by immunoassay. Commercially available ELISA kits were used for the detection of human IL-12p70 and mouse IFNγ (BD Biosciences).

### Tumor cell lines

HT29 and CT26 tumor cell lines were obtained from American Type Culture Collection (Rockville, MD, USA).

### Elicitation of DC maturation by *E Coli* associated to neutrophils

2×10^5^ human neutrophils were incubated with heat-inactivated *E. coli* bacteria in various numbers ranging from 10^5^–10^2^ for 60 min. The neutrophils were then washed eight times and the pellets and the supernatants collected. Washed PMN and the supernatants were added to human immature DC (iDC) cultures and 48 h later IL-12 concentrations in the supernatants were monitored. Subsequently, such DC (10^5^) were washed in cold PBS and incubated 15 min at 4°C with specific FITC or PE-labeled mAbs to measure the mean fluorescence intensity for CD80, CD83 and CD86 surface immunostaining as assessed by flow cytometry. Mature DC (mDC) in the presence of poly I:C, TNFα and IFNα were used as a positive control.

### OVA-specific T cell response analysis

Splenocytes from OT-1 and OT-2 transgenic mice were stained with CFSE solution 2.5 µM. After 24 h co-culture DC plus live or UV-irradiated neutrophils (cultured during 2 h with or without OVA at 1 mg/ml), OT-1 or OT-2 splenocytes were added to the co-culture. The capacity of DC to cross-present OVA from neutrophils was measured as the dilution of membrane CFSE from CD4^+^ (OT-2 mice) or CD8^+^ (OT-1mice) splenocytes that were gated by immunostaining with CD8 or CD4 specific mAb in a FACSCalibur.

### OVA protein electroporation in the tumor cell line CT26

For electroporation, CT26 (3.5×10^6^ cells/500 µl RPMI without serum and antibiotics) were incubated with 500 µl OVA at 2 mg/ml for 10 minutes at room temperature. Electroporation was carried out using Gene Pulser® II Electroporation System (BioRad, Hercules, CA, USA) using the conditions of two pulses at 200 V/cm and 300 µF of capacitance. Immediately after electroporation, CT26 cells were cultured in RPMI complete medium during at least 1 h. To ensure the internalisation of OVA in CT26 cells, a control OVA fluorescein conjugate (Invitrogen) was electroporated and its internalisation was assessed by flow cytometry after extensive washing of non internalized protein.

### Co-injection of DC and PMN into the mouse foot pad

Mouse PMNs were co-cultured for 2 h with 10^5^ heat-inactivated *E. Coli* bacteria. After 8 extensive washes, 5×10^6^ PMN-PKH26 and 10^7^ mouse DC-PKH2 were injected into the foot pads of mice in separate syringes with 30 µl PBS for each injection. 24 h later, popliteal and inguinal lymph nodes were surgically excised and were disaggregated to give single cell suspensions. Cells were immunomagnetically selected to enrich CD11c^+^ cells. Immunoselected cells were processed for subsequent analysis using flow cytometry and confocal microscopy.

### Statistics

All data are presented as the mean ± SD. For comparisons, unpaired Student's t-tests or Mann–Whitney U tests were used. Calculations were made using Prism software (Graph Pad Software, La Jolla, CA, USA).

## Results

### DC and PMN are co-attracted by IL-8 derived from tumor cells

**Figure 1:**
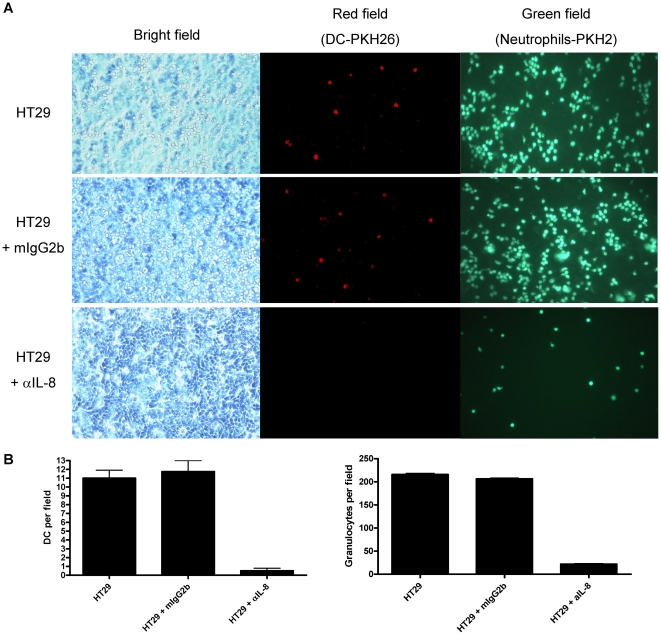
DC and PMNs are co-attracted by IL-8 secreted by HT29 colon cancer cells. (A) Chemotactic migration of DC (labeled with PKH26) and PMN (labeled with PKH2) from the upper chamber towards confluent monolayers of HT29 cells in the lower chamber. When indicated, control IgG mAb or anti IL-8 mAb were added to the lower chamber. Microscopy photographs represent visible light and UV illumination fields with filters for red and green fluorescence showing the lower chamber when taken 24 h later. (B) Quantitative data based on experiments as in A, representing three independent triplicate experiments counting four microscopic fields per well (mean±SD).

We have previously shown that human carcinoma cells produce functional IL-8 [32]. One of the corollaries is that such IL-8 might attract human PMNs and monocyte-derived DC [27]. Indeed, in classical transwell chemotaxis assays towards IL-8-producing monolayers of HT-29 human colon carcinoma cells, both PKH26-labelled DC and PKH2-labelled PMNs are actively co-attracted to the lower chamber containing a confluent monolayer of HT29 cells (figure 1A and B). The effect was mediated by IL-8 as demonstrated by the abrogation of the migration by adding a neutralizing anti-IL8 mAb to the lower chamber (Figure 1A and B).

### DC take up live and UV-irradiated PMN leukocytes that might carry microbial maturation signals

**Figure 2:**
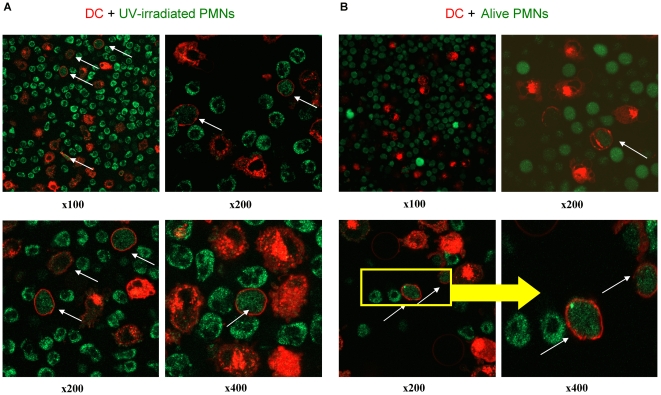
Human DC internalize UV-irradiated and viable PMNs. Confocal microscopy images at the indicated magnifications of 4 h co-cultures of human DC (membrane-red labeled with PKH26) and either (A) peripheral blood PMNs lethally irradiated with UV (green labeled with PKH2) or (B) identical viable PMNs. White arrows indicate a DC with internalized PMN-derived material.

Human DC and PMN were florescence-labelled and as can be seen in figure S1, fluorescence was readily observed by flow cytometry and confocal microscopy. In figure 2 confocal microscopy pictures of short 4h co-cultures of PMN and DC show that fluorescent material from human PMNs was internalized by human DC (Figure 2). Analyses of multiple experiments indicated in confocal microscopy planes that DC-red fluorescence was surrounding PMN-green fluorescence but the contrary was not observed.

PMN internalization by DC occurred both when the PMN had been irradiated with UV-light or when live untouched PMNs were used. UV irradiation was used to induce cell death in PMNs (Figure S2), as they sequentially acquire staining by annexin-V and 7-AAD when undergoing apoptosis. Evidence for internalization in confocal planes is shown at different magnifications and summarized in a video reconstruction of multiple confocal planes in the Z axis (Video S1).

**Figure 3:**
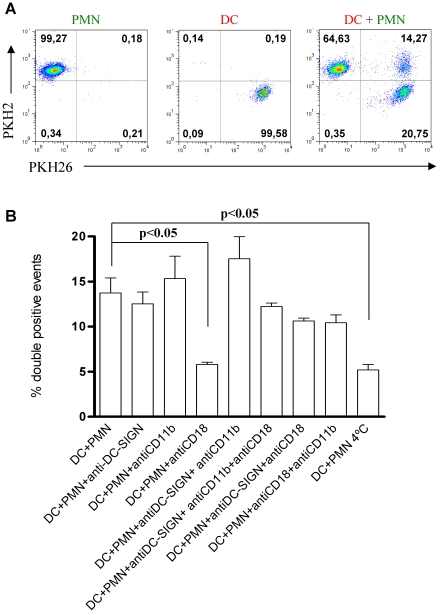
Human DC internalize PMNs in a temperature and CD18 dependent fashion. (A) FACS dot plots showing PKH26 staining of Human DC and PKH2 staining of PMNs and 4 h co-cultures of DC and PMN showing double positive events in a representative experiment of at least five similarly performed. (B) Co-culture experiments as in A, depicting the percentage of double positive events at 4 h of co-culture. When indicated blocking mAb to DC-SIGN, CD11b and CD18 were added or the co-cultures were performed at 4°C.

In figure 3A a two color FACS dot plot shows the single stained cultures and the double positive events corresponding to DC that have internalized green-fluorescent material from PMNs. Although the FACS technique cannot rule out aggregation as the cause for double positive events these data in conjunction with confocal microscopy indicate that most double positive events are explained by internalization.

Using this technique, we looked at the requirements for internalization. We observed that neither an anti-DC-SIGN antibody not and anti-CD11b antibody were able to decrease internalization (Figure 3B). It is of note that such adhesion molecules have been described as the main mediators of the DC-neutrophil heterotypic aggregation [20]. In contrast, anti-CD18 integrin common chain mAb clearly decreased double staining. When the experiment was performed at 4°C to repress metabolism, internalization was drastically reduced (Figure 3B).

**Figure 4:**
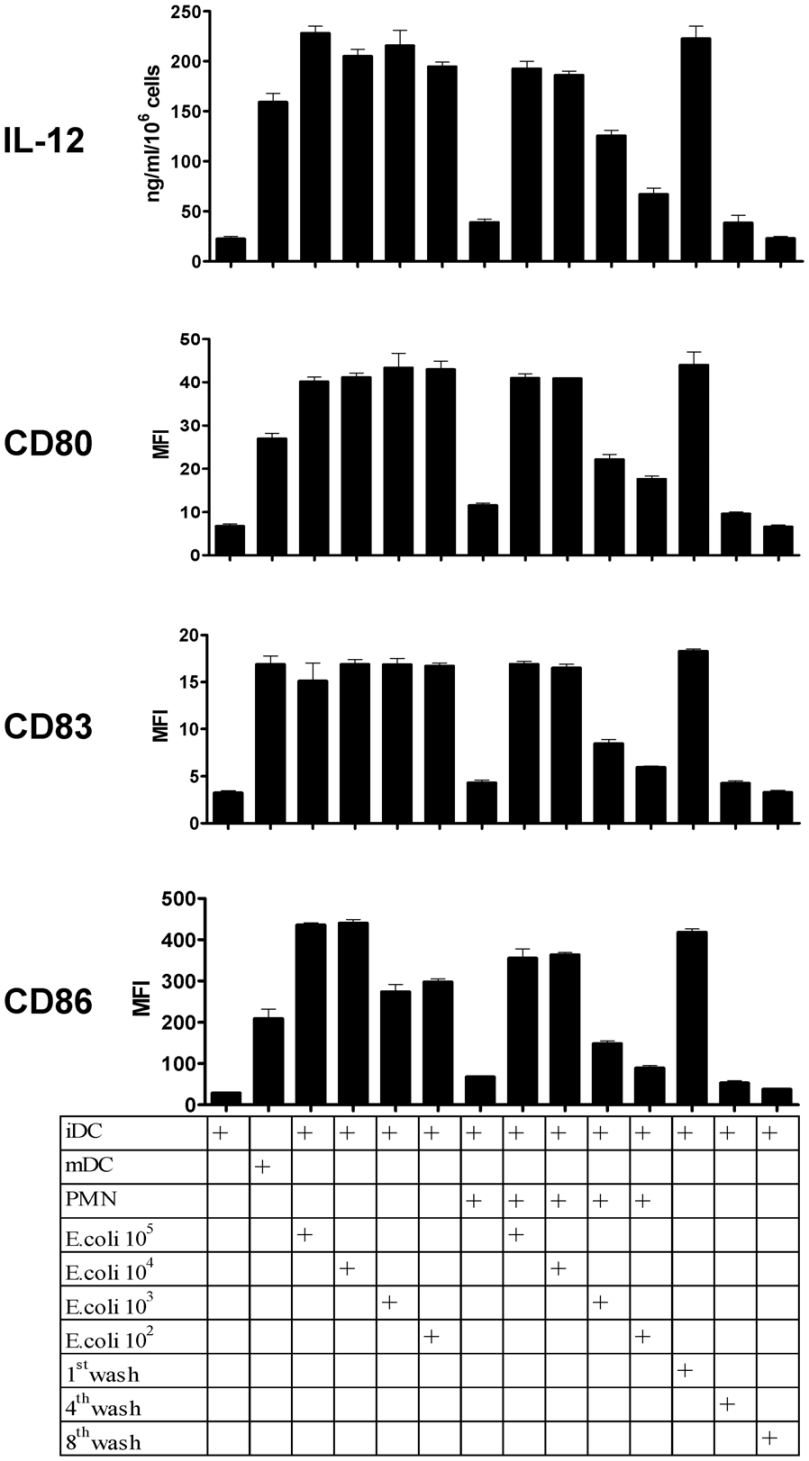
PMNs carrying *E. coli* bacteria trigger the maturation of DC. PMN from human peripheral blood were exposed for 1 h to *E. coli* bacteria for 60 min in various numbers ranging from 10^5^–10^2^ and then were washed eight times and the pellet and the supernatant collected. Washed PMN and the indicated washing supernatants were added to human immature DC (iDC) cultures and 48 h later IL-12 concentration in the DC culture supernatants was monitored as well as the mean fluorescence intensity for CD80, CD83 and CD86 surface immunostainings on the DC. Mature DC (mDC) in the presence of poly I:C, TNFα and IFNα were used as a positive control.

If PMN have internalized bacteria they are likely to contain microbiological biomolecules that activate/mature DC (i.e: LPS or bacterial DNA). In fact, we exposed PMNs to *E. coli* bacteria which were rapidly internalized (Figure S3). We removed bacteria from cells by extensive washing and centrifugation at low speed (up to 8 washing cycles with 50 ml of culture medium each). PMN that had been exposed to *E. coli* were able to mature DC in terms of IL-12 production and enhancement of CD80, CD83 and CD86 surface expression. The maturation activity was only present in the cellular pellets since the supernatant of the eighth wash was not able to trigger DC maturation in any case (Figure 4). These findings are important since extravasated PMN that would capture gram negative bacteria would concentrate LPS and bacterial DNA for a subsequently interacting DC.

### Internalization of mouse bone marrow PMN by mouse DC

**Figure 5:**
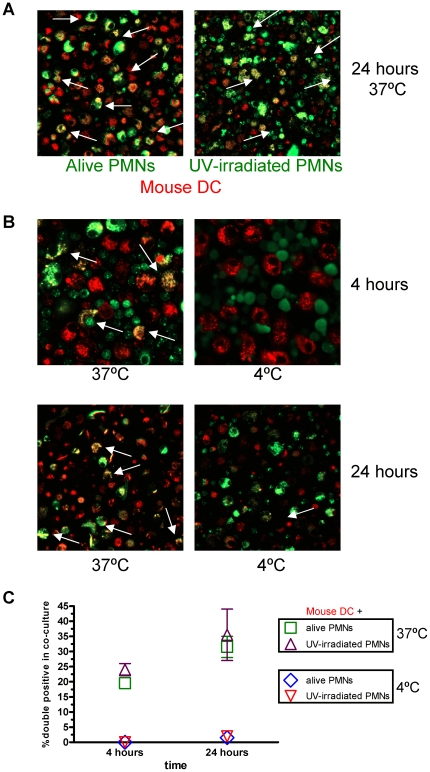
Mouse DC take up mouse PMN leukocytes. (A) Confocal microscopy images of 24 h co-cultures of viable or UV-irradiated PMNs labeled with PKH2 and bone-marrow derived DC labeled with PKH26. Arrows show co-localization of both dyes. (B) Similar experiments as in A but with some co-cultures performed at 4°C as indicated. (C) Summary of data from five microscopic fields per condition in which internalization was identified by yellow staining.

To study the consequences of PMN internalization by DC, it was of interest to move on to mouse experimental systems. Consequently, we purified Ly-6G cells from bone marrow cells by immunomagnetic selection (Figure S4A) and DC were derived from bone marrow in the presence of GM-CSF for 9 days. Figure S4A shows immunostainings for Gr-1 (an antibody that recognizes both Ly-6C and Ly-6G) and Giemsa stainings of cytospins from the enriched PMN population. Mouse DC and PMNs were also differentially labelled with fluorescent dyes. Co-cultures of DC and PMN for 4 h showed evidence of internalization by flow cytometry (Figure S4B) and confocal microscopy (Figure S4C and figure 5A, at 24 h of co-culture). Pictures in figure 5B show confocal microscopy evidence in the sense that internalization of PMN by DC is inhibited at 4°C both in 4 h and 24 h co-cultures. Summarized data are presented in figure 5C. Therefore the phenomena observed with human DC and PMN seems to be similar in both humans and mice.

### Dead carcinoma cells are internalized by PMN leukocytes that if subsequently phagocytosed by DC ferry carcinoma cell material

**Figure 6:**
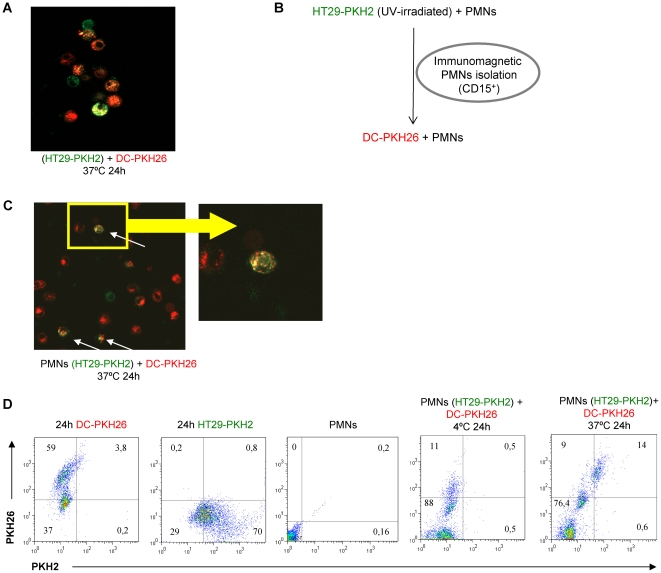
Fluorescent material from human carcinoma cell debris can be taken up by PMN and transferred to DC. (A) PKH2-labeled HT29 cells were UV-irradiated and co-cultured for 24 h with human PKH26-labelled DC and confocal images were taken indicating internalization by DC. (B) Summary of the experimental approach of co-cultures consisting of HT29 carcinoma cells previously labeled with PKH2 and UV-irradiated with PMNs which were subsequently retrieved 2 h later by CD15 immunomagnetic selection. Then PMN were co-cultured for 24 h with PKH26-labeled DC. (C) Confocal microscopy images of the indicated co-culture conditions. (D) FACS dot plots showing each cell type labeled with the corresponding fluorescent dye and co-cultures performed at 37°C and 4°C.

Human DC can take up fluorescent material from apoptotic tumor cells [33]. We confirmed this point in figure 6A that shows that red-labelled DC can take up green fluorescent apoptotic bodies from UV-irradiated HT29 colon carcinoma cells (Figure 6A). Apoptotic tumor cells can be prey of PMN phagocytes as well. We reasoned that if PMN leukocytes take up carcinoma cell debris, this material will end up inside DC, in the case that such DC eventually internalized a PMN with HT29 material inside. To test this idea we labelled HT29 colon carcinoma cells with PKH2 and following UV-irradiation fed them to PMN leukocytes. After 2 h of co-culture, CD15^+^ cells were immunomagnetically selected to re-isolate the PMNs. Subsequently CD15^+^ PMNs were co-cultured with PKH26-labelled DC for 24 hours. The experimental approach is summarized in figure 6B. As can be seen in figure 6C, green fluorescent material from HT29 cells is found inside red-labelled DC. Furthermore, quantitative data from FACS analysis indicated that the internalization phenomenon is quite efficient with more than half of the DC taking up green fluorescent material derived from the carcinoma cells (Figure 6). Such internalizing activity was abolished if the co-incubation of PMN and DC took place at 4°C.

Accordingly it could be envisaged that PMN exposed to debris from tumor cells could transfer this debris to third party antigen-presenting DC. However, to explore the role in antigen cross-presentation we needed to use mouse models.

### Antigen carried by PMN can be cross-presented by DC *in vitro*

**Figure 7:**
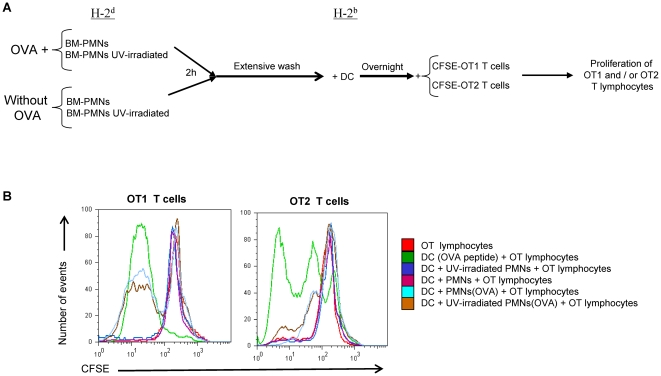
Ovalbumin taken up by mouse PMNs can be transferred to DC that in turn cross-present antigen. (A) Schematic representation of the experimental conditions in which OVA was added to bone-marrow-derived PMNs from BALB/c mice which were extensively washed 2 h later and subsequently subjected or not to UV-irradiation. Then PMNs were co-cultured overnight with DC derived from the bone marrow of C57BL/6 mice. The next morning CFSE-loaded OT-1 or OT-2 TCR-transgenic T cells were added to the cultures. (B) FACS histograms showing CFSE dilution at 72 h of culture in the indicated conditions. DC pulsed with the cognate OVA peptides recognized by OT-1 and OT-2 T lymphocytes were used as positive controls. The experiment shown is representative of at least three similarly performed.

In order to demonstrate that DC could cross-present antigen material from internalized PMNs, we performed experiments with bone-marrow neutrophils derived from the bone marrow of BALB/c mice (H-2^d^) from which PMN were isolated as shown in figure S4A. Such PMN were given OVA protein for two hours in culture followed by extensive washes (x 8 times). These PMNs were then co-cultured overnight with DC from C57BL/6 mice (H-2^b^) and exposed to OT-1 or OT-2 TCR-transgenic lymphocytes. Proliferation of OT-1 or OT-2 T cells was monitored by CFSE dilution 72 h later. An schematic representation of the approach based on the fact that H-2^d^ class I and II antigen presenting molecules cannot present to OT-1 or OT-2 T lymphocytes is presented in figure 7A. In figure 7B (overlay histograms) and figure S5 (individual histograms separated for clarity), a representative case shows the dilution of CFSE that was triggered by the cognate peptides pulsed on DC as a positive control and by DC that had been incubated with PMNs preloaded with OVA. T cell division did not take place in the rest of control conditions including PMN that had not been exposed to OVA fed to DC (Figure 7B and figure S5). Confirmatory independent experiments offered similar results (Figure S6A), that were consistent with analyses of IFNγ concentrations released to the tissue culture supernatants (Figure S6B). As a whole these data indicate that OVA antigen determinants are cross-presented by the DC which take up antigen from PMN at least in the mouse system.

### Carcinoma cells loaded with OVA transfer antigen to PMNs, then PMNs to DC and such DC cross-present antigen to T lymphocytes

**Figure 8:**
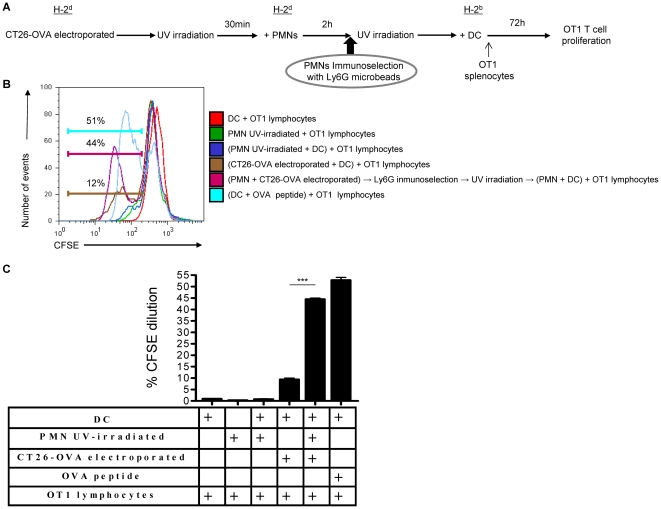
Internal antigens can be ferried from tumor cells to DC via PMNs and then be subsequently cross-presented to T lymphocytes. (A) Schematic representation of the experiments in which CT26 tumor cells were electroporated in the presence or not of soluble OVA protein, extensively washed 30 min later and then UV-irradiated. Such apoptotic tumor cells were co-cultured with Ly-6G-immunoselected PMN from the bone marrow of BALB/c mice (H-2^d^) during 2 h. These PMN that kept labeled with the immunomagnetic beads were re-isolated in columns under a magnetic field and subsequently exposed to DC from C57BL/6 mice, whose H-2K^b^ antigen presenting molecules can present antigen to OT-1 T cells. Following overnight DC culture with the PMNs, CFSE OT-1 lymphocytes were added to the cultures and CFSE dilution in CD8 cells was monitored 72 h later. (B) Representative histograms of CFSE dilution in the indicated conditions including DC pulsed with the cognate peptide of OT-1 T cells as a positive control. (C) Summary (mean±SD) of three experiments similarly performed. Asterisks indicate statistical significance p<0.001 in student's t test.

In order to study antigen transfer to DC via a PMN intermediate, experiments were set up as summarized in figure 8A. CT26 colon carcinoma cells of BALB/c origin (H-2^d^) were electroporated in the presence of soluble OVA protein. Electroporation in the presence of a soluble protein leads to internalization of protein as checked with OVA-FITC. Figure S7 shows internalization of FITC fluorescence by CT26 contingent on the electrical shock. Once CT26 had been electroporated with or without OVA, they were extensively washed, UV-irradiated and fed to PMN of BALB/c origin. Such PMN were recovered 2 h later from the cultures by Ly-6G magnetic immunoselection (taking advantage of the magnetic beads still bound to the cells). Immunoselected PMNs were then co-cultured overnight with DC of C57Bl/6 (H-2^b^) origin. Subsequently these DC were used to stimulate CFSE-loaded OT-1 T lymphocytes. As can be seen in a representative experiment shown in figure 8B, dilution of OT-1 CFSE only took place when the CT26 tumor cells had been electroporated with OVA, but not in the remaining control co-cultures. As a positive control DC pulsed with the cognate peptide were used and elicited strong proliferation of OT-1 lymphocytes. This correlated with IFNγ release to the co-culture supernatant (data not shown).

These data indicate that it is possible that tumor derived antigens can end up productively presented by DC to T cells after being transiently ferried by PMNs that are internalized by the DC.

### DC co-injected in the foot pad with PMNs that have internalized bacteria reach draining lymph nodes with PMN-derived material

**Figure 9:**
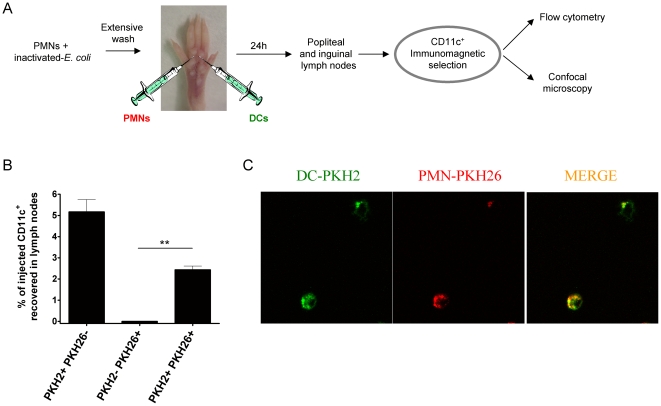
Co-injection of PMNs with endocytosed bacteria and DC in subcutaneous tissue gives rise to the detection of DC carrying PMN-derived material in the draining lymph node. (A) Schematic representation of experiments in which bone marrow-derived DC and PMNs that had been pre-exposed to *E. coli* and washed were injected in separate syringes into the foot pads of mice. Both leukocyte types were differentially labelled with fluorescence dyes (PKH26 for PMNs and PKH2 for DC). (B) Calculations of the percentages of injected DC recovered as CD11c^+^ immunomagnetically selected cells from popliteal lymph nodes measuring double or single positive fluorescent events as indicated by flow cytometry. (C) Representative confocal images of cytospins made with the CD11c-immunoselected cells from popliteal lymph nodes. Experiments are representative from at least two similarly performed with three mice per group each. Asterisks indicate statistical significance p<0.01 in student's t test.

To present antigen to T cells DC need to reach lymph nodes through afferent lymphatic vessels [34]. This is a function tightly controlled upon maturation of the DC by the induction of CCR7 expression [17], [19]. If DC and PMN are injected in the foot pad they should be able to meet thus mimicking inflamed tissue. In the experimental approach shown in figure 9A, differentially fluorescence labelled bone marrow derived DC and PMN preloaded with *E. coli* bacteria were injected with separate syringes into the foot pad. 24 h later draining lymph nodes were surgically excised and CD11c cells enriched by immunomagnetic selection. Double immunofluorescence events analyzed by flow cytometry and summarized in figure 9B indicate that about 2% of the injected DC were recovered from the draining LN carrying fluorescent material from the PMNs while about 5% of the injected DC were recovered from the lymph nodes with no evidence of carrying PMN-derived fluorescent material. These data indicate that is possible for DC which internalize PMN material to reach T cell compartments. It is of note that if PMN had not been exposed to bacteria very few CD11c^+^ fluorescent events were recovered from lymph nodes and virtually no double positive cells were found (data not shown). Confocal microscopy images of cytospin preparations made with CD11c^+^ magnetically-selected cells from draining lymph nodes indicated co-localization of fluorescent dyes in the same cells.

**Figure 10:**
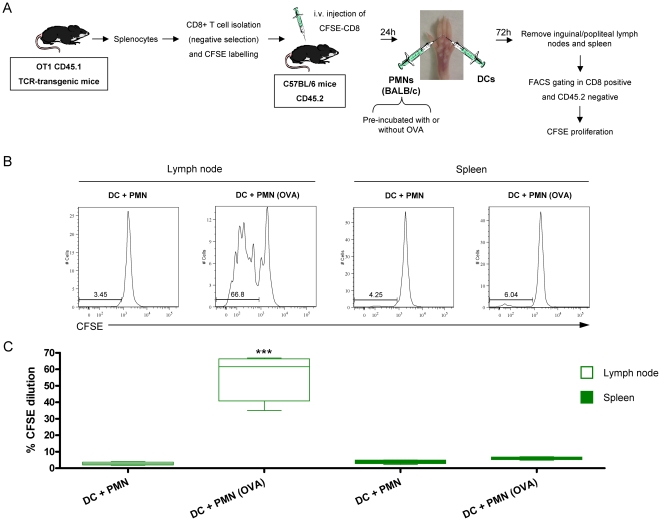
Cross-presentation of antigens taken by DC from PMNs take place *in vivo*. (A) Schematic representation of experiments in which splenocytes from OT1 CD45.1 TCR-transgenic mice were subjected to CD8^+^ T cell isolation by immunomagnetic negative selection. OT-1 Cells were labeled with CFSE and 2×10^6^ were injected intravenously into C57BL/6 mice. 24 h later mice received injections of PMNs and DC (H-2^b^) in the footpad. Immunomagnetically isolated PMN were from BALB/c mice (H-2^d^) and were pre-incubated or not with OVA for two hours and extensively washed. PMNs and DC were injected with different syringes in the footpads of the mice which had been transferred with CFSE-labelled OT-1 cells 24 hours before. 72 h later proliferation of OT1 CD45.1^+^ cells was assessed by flow cytometry in draining lymph node and spleen (B) Representative histograms of CFSE dilution seen in lymph nodes and spleen. (C) Summary data of CFSE expression from lymph nodes and spleens in 3 experiments performed as described in A. When indicated PMN were pre-incubated or not with OVA. Asterisks indicate statistical significance p<0.001 in Mann-Whitney U test.

Next we examined if cross-presentation of antigens taken by DC from PMNs could take place in vivo. For this purpose H-2^b^ DC and H-2^d^ neutrophils were injected in different syringes in the footpad of C57BL/6 mice. PMNs had phagocytosed OVA and thoroughly washed for eight times as described in figure 10A. Mice had received 24h prior to the experiment CFSE-labelled OT-1 cells to monitor their proliferation. As shown in figures 10B and C, OT-1 lymphocytes only proliferated when the mice had been injected with PMNs which had phagocytosed OVA. Since PMNs cannot directly present antigen to OT-1 T lymphocytes it is concluded that it is the independently injected DC present antigen. It is of note that when injecting OVA-loaded H-2^d^ PMN without DC a certain level of OT-1 proliferation was observed (25–35%) that can be attributed to endogenous antigen presenting cells (data not shown).

## Discussion

In the defence of the organism against infection it makes sense that the early innate response would cue the adaptive immune responses. In the bridge between innate and adaptive immune responses dendritic cells play a pivotal role [35]. We reasoned that pyocytes (neutrophils forming pus) would be an excellent source of concentrated microbial antigens and microbial molecular pattern signals, if eventually internalized by DC.

Therefore we first found that both human and mouse DC avidly internalize PMNs in co-culture. Both apoptotic and viable PMN are internalized although with more efficiency in the case of apoptotic PMN. It is postulated that under steady state conditions this phenomenon should feed DC with innocuous self antigens that if presented by the immature DC should lead to tolerance [36].

The nature of the surface molecules playing a role in the internalization is unclear. We found partial inhibition by blocking the CD18 integrin chain but not upon blockade of DC-SIGN and CD11b in many attempts. This is in disagreement with a paper from the group of Van Kooyk whose findings indicated a role for DC-SIGN on DC and sialyl Lewis-X decorating CD11b in the heterotypic cellular interaction [20], [21]. We observe blockade by a monoclonal antibody binding the common leukocyte integrin β chain, while CD11b seems not to be involved. Therefore a careful re-examination of the surface molecules dynamically involved in the DC-neutrophil interactions is needed and is the subject of current experiments.

Since internalization rather than aggregation is our read out, reasons for this difference could come from various sources. Internalization requires active metabolism since it was nearly abolished at low temperatures.

Under acute inflammation, extravasated PMN would be full of phagocytosed microbes and microbial material. An important aspect is that such pyocytes, if entering into contact with and become internalized by DC, would transmit to DC both the presence of the biomolecules denoting a microbial pathogen and microbial antigens. In our proof of concept experiments, *E. coli* bacteria actively loaded into PMN was able to intensely mature the subsequently encountered DC. This makes sense because ingestion of PMN by DC or their tissular precursors will launch the proper maturation program in DC. In mice these events also elicit migration of DC to draining lymph nodes. It remains to be seen which molecular patterns of bacteria are detected, but most likely these involve LPS detection by TLR4 and bacterial DNA detection by TLR9 in our experiments.

DC have been in the limelight of tumor immunology and immunotherapy [37]. In solid tumors multiple myeloid cells nest in the stroma including cells resembling PMN. Such PMN have the opportunity to phagocytose apoptotic or necrotic tumor cells. We thought that it was interesting to determine if PMN could take up antigen from carcinoma cells and then transfer it to DC, in such a way that the relevant antigens would end up cross-presented to T cells. To start exploring these possibilities we moved to mouse systems in which we can probe for antigen presentation with TCR-transgenic T cells. Our data in humans strongly argued in favour of this possibility because HT29 fluorescent carcinoma cell debris taken up by PMNs is internalized by DC, if those PMN are subsequently phagocytosed. However, it could be argued that the PMN-associated fluorescent material could be degraded and is thereby become not suitable for antigen presentation. However, in the mouse system we found that PMN which had internalized OVA protein from soluble sources or OVA-electroporated tumor cells efficiently cross-presented antigens to either OT-1 or OT-2 lymphocytes. Direct presentation by PMN or tumor cells was ruled out because of incompatible antigen presenting molecules although in other circumstances this can take place according to published reports [24], [38]. There is a tendency in our hands to observe more proliferation induced in this setting on OT-1 than on OT-2 cells and further studies should clarify the relative efficiency of antigen presentation via class I and class II when antigen is acquired from PMNs.

The study of the intercellular crosstalk between DC and PMN leukocytes is in its infancy. A recent report indicates that PMN could deactivate DC upon arrival to lymph nodes in aseptic conditions [39]. This makes sense for immature DC but it remains to be seen if it is the case *in vivo* under septic conditions, that ought to be more relevant for the defence of the body against infection. Our data showing the ability of DC loaded with material derived from PMN with internalized bacteria to reach the lymph nodes argue in favour of DC-maturing role of PMNs under acute infection.

Our in vivo results in mice indicate that DC can cross-present antigen transferred from a PMNs to resting CD8+ T cells. In our experimental setting it is likely that DC in the footpad take-up antigen and migrate to lymph nodes where they present antigen to T cells. Interestingly there is indication that endogenous DC can also mediate this effect (Alfaro C. *et al.*, unpublished results).

Our current research is focused on unravelling the implications of these PMN-DC interactions by carrying out *in vivo* experiments in mouse models. In particular our data should be interpreted with caution since we demonstrate these phenomena with DC differentiated in culture, but not yet with the endogenous DC.

In humans these events are much more difficult to dissect *in vivo*. However, there is published evidence of DC-PMN interactions in appendicitis surgical samples [20], [22] and we have found that DC and PMN can be co-attracted to solid malignancies by means of the IL-8 chemokine.

All in all, our experiments point to an entangled and complex relationship between PMN and DC. PMN greatly outnumber DC and therefore could act as concentrators of antigen and microbial molecules for DC. If PMN leukocytes become accessible to DC they can be internalized and then modulate DC functions while transferring the antigens that PMN may carry. If these functions are mediated by endogenous DC as we observe with DC differentiated in culture, these can be important phenomena. However, exactly how relevant are these functions for the overall physiology of the immune system still remains to be seen.

## Supporting Information

Figure S1 Labeling of human PMN and DC with the indicated fluorescent dyes visualized by FACS and by confocal microscopy.
(TIF)

Figure S2 Labeling of human PMNs undergoing or not UV-irradiation with Annexin V and 7-AAD visualized by FACS. The percentages of each quadrant indicate the differences between alive PMNs and UV-irradiated PMNs.
(TIF)

Figure S3 Microscopic GRAM-stained images (x 400 magnification) of *E. coli* bacteria and PMNs exposed to *E. coli* bacteria at time 0 and 2 hours later. Red circles indicate small group of *E. coli* bacteria.
(TIF)

Figure S4 Purification of mouse neutrophils and co-cultures with mouse DC. (A) Immunomagnetic selection of Ly-6G cells from single cell suspensions from mouse femur and tibia bone-marrow before and after immunomagnetic selection (Automacs^®^). Purity was assessed by cells showing bright Gr-1 immunostaining and upon May-Grünwald Giemsa stainings of cytospins. (B) FACS dot plot analysis of mouse DC and PMN labeled with PKH2 and PKH26 as a single cell type or in co-culture as indicated. 4 h co-cultures were performed at 37°C or 4°C as indicated. (C) Representative confocal images of co-cultures set up with the PMN and DC at the indicated temperature conditions.
(TIF)

Figure S5 Individual histograms from the overlay shown in figure 7B. Histograms and graphs indicate dilution of CFSE that was triggered by the cognate peptides pulsed on DC as a positive control and by DC that had been incubated with PMNs pre-loaded or not with OVA.
(TIF)

Figure S6 CFSE dilution and IFNγ concentrations of OT-1 and OT-2 cell cultures (A) Shows the extent of CFSE dilution in OT-1 and OT-2 cells. In a replicate experiment performed identically to the one shown in figure 7B. (B) IFNγ concentrations released in 72 h and 96 h to the supernatant of cultures by OT-1 and OT -2 lymphocytes in conditions identical to those in figure 7 (as indicated in the figure) in the same experiment in which CFSE dilution was monitored in A.
(TIF)

Figure S7 Dot plots FSC/SSC and green fluorescence (FL1) of CT26 tumor cells set in the presence of OVA-FITC and electroporated or mock-electroporated as indicated. Cells were analyzed by flow cytometry for FITC fluorescence 2 h following electroporation or mock electroporation.
(TIF)

Video S1 Video reconstruction of multiple confocal planes in the Z axis.
(MPG)
