# Supplementary figures and images for: Dendritic Cells Take up and Present Antigens from Viable and Apoptotic Polymorphonuclear Leukocytes
Source: PLoS One. 2011 Dec 20;6(12):e29300. doi: 10.1371/journal.pone.0029300 (PMC3243708; doi:10.1371/journal.pone.0029300)

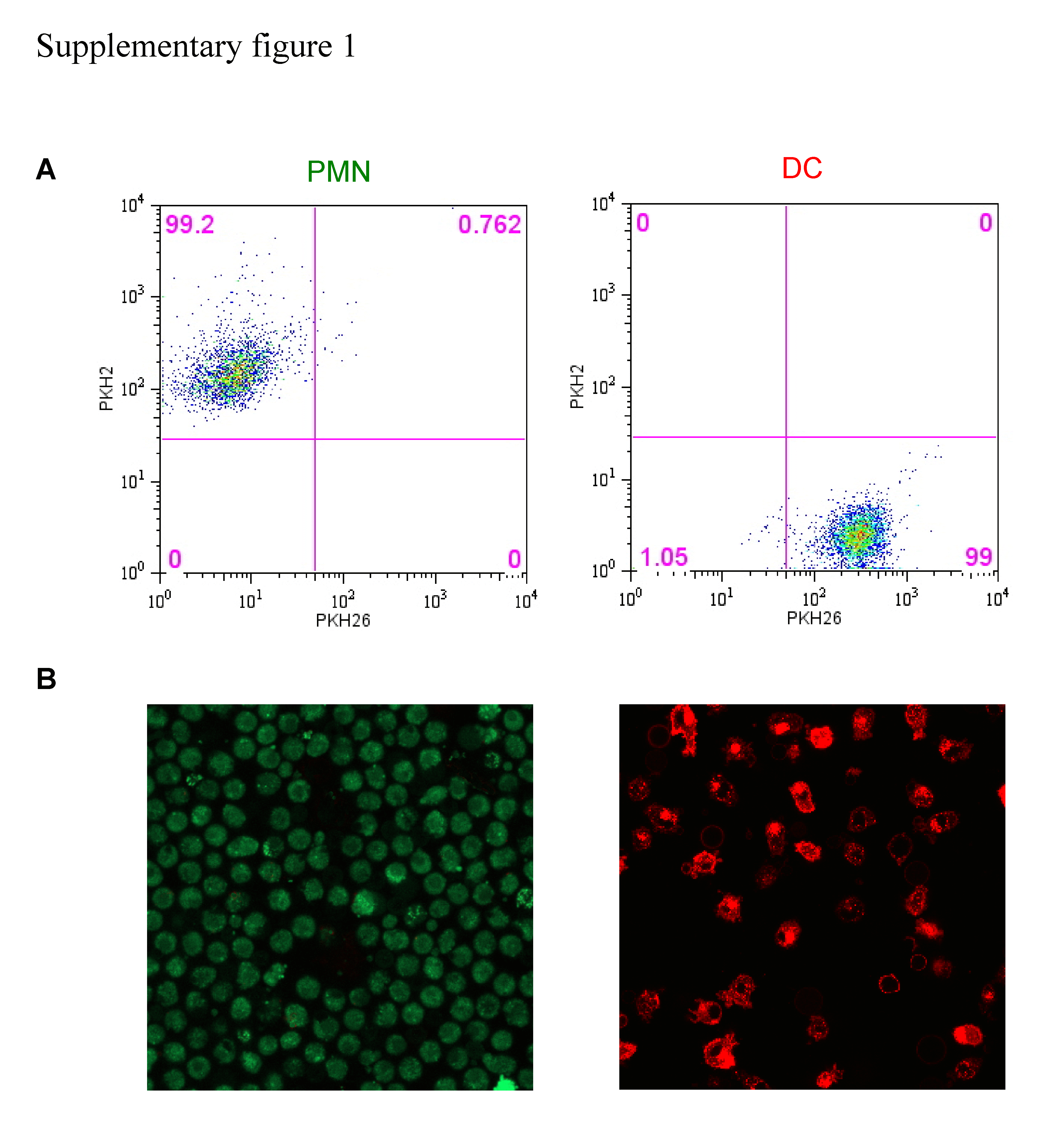

Supplement: Figure S1 — Labeling of human PMN and DC with the indicated fluorescent dyes visualized by FACS and by confocal microscopy. (TIF) [file pone.0029300.s001.tif]

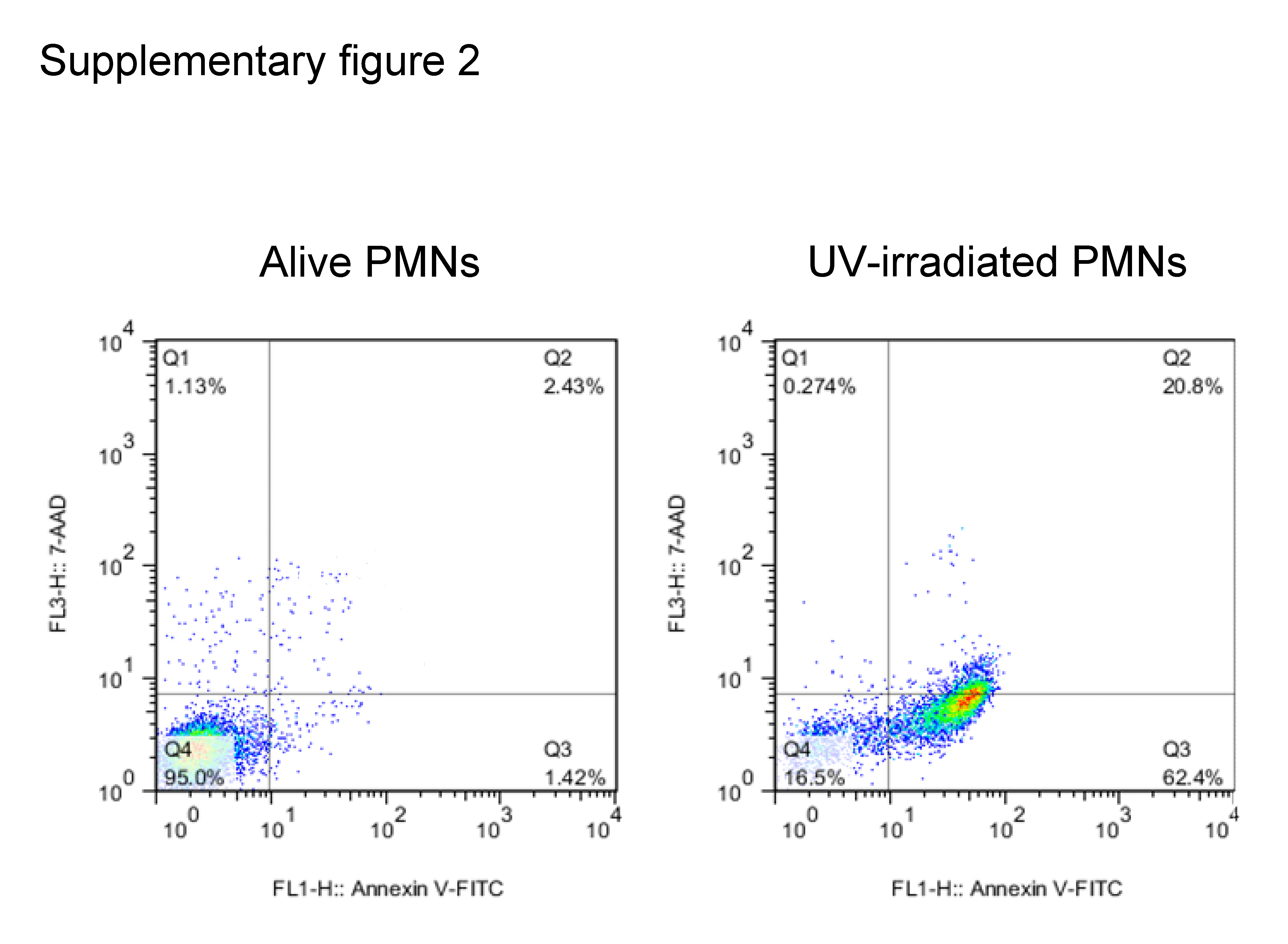

Supplement: Figure S2 — Labeling of human PMNs undergoing or not UV-irradiation with Annexin V and 7-AAD visualized by FACS. The percentages of each quadrant indicate the differences between alive PMNs and UV-irradiated PMNs. (TIF) [file pone.0029300.s002.tif]

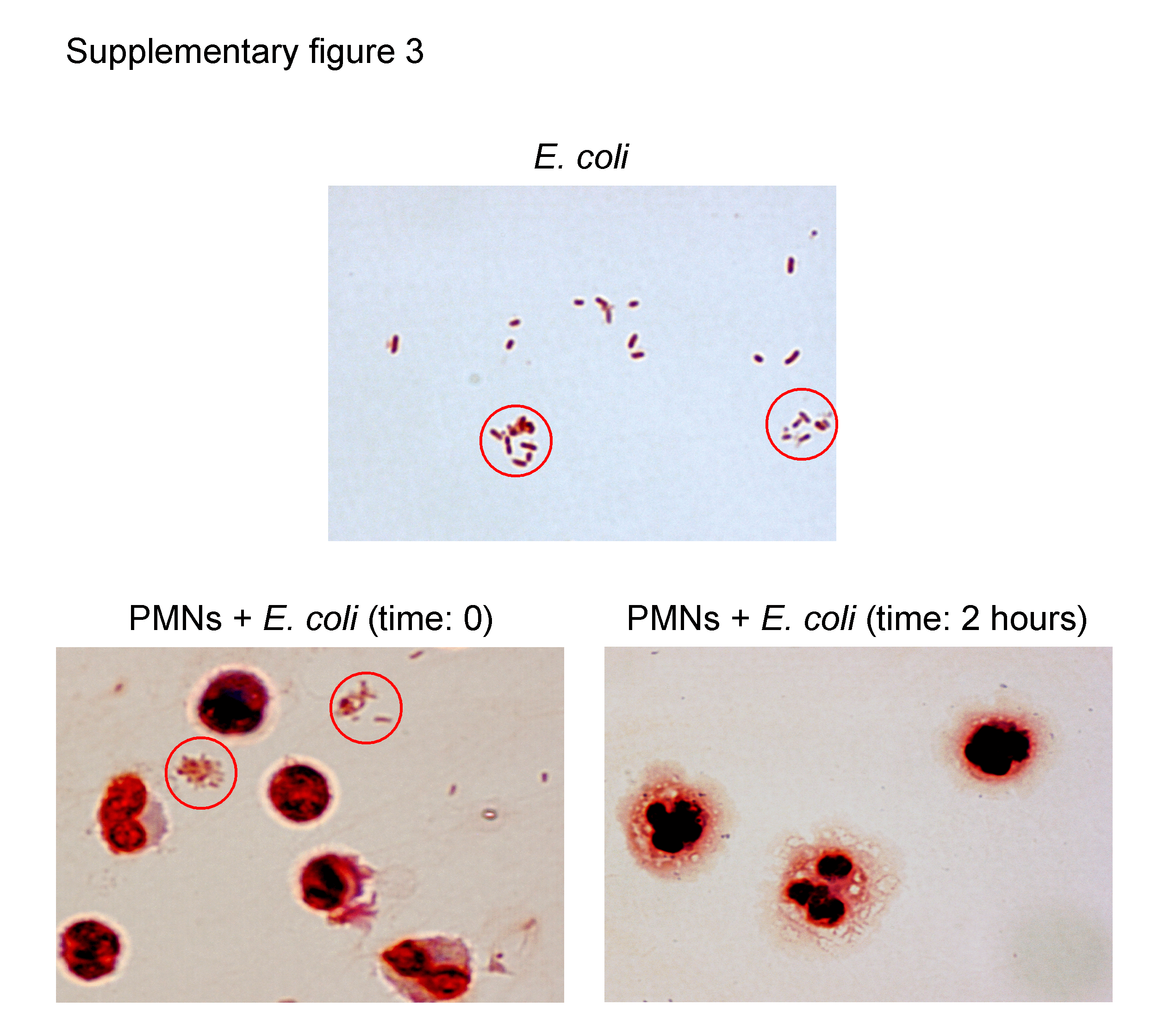

Supplement: Figure S3 — Microscopic GRAM-stained images (x 400 magnification) of E. coli bacteria and PMNs exposed to E. coli bacteria at time 0 and 2 hours later. Red circles indicate small group of E. coli bacteria. (TIF) [file pone.0029300.s003.tif]

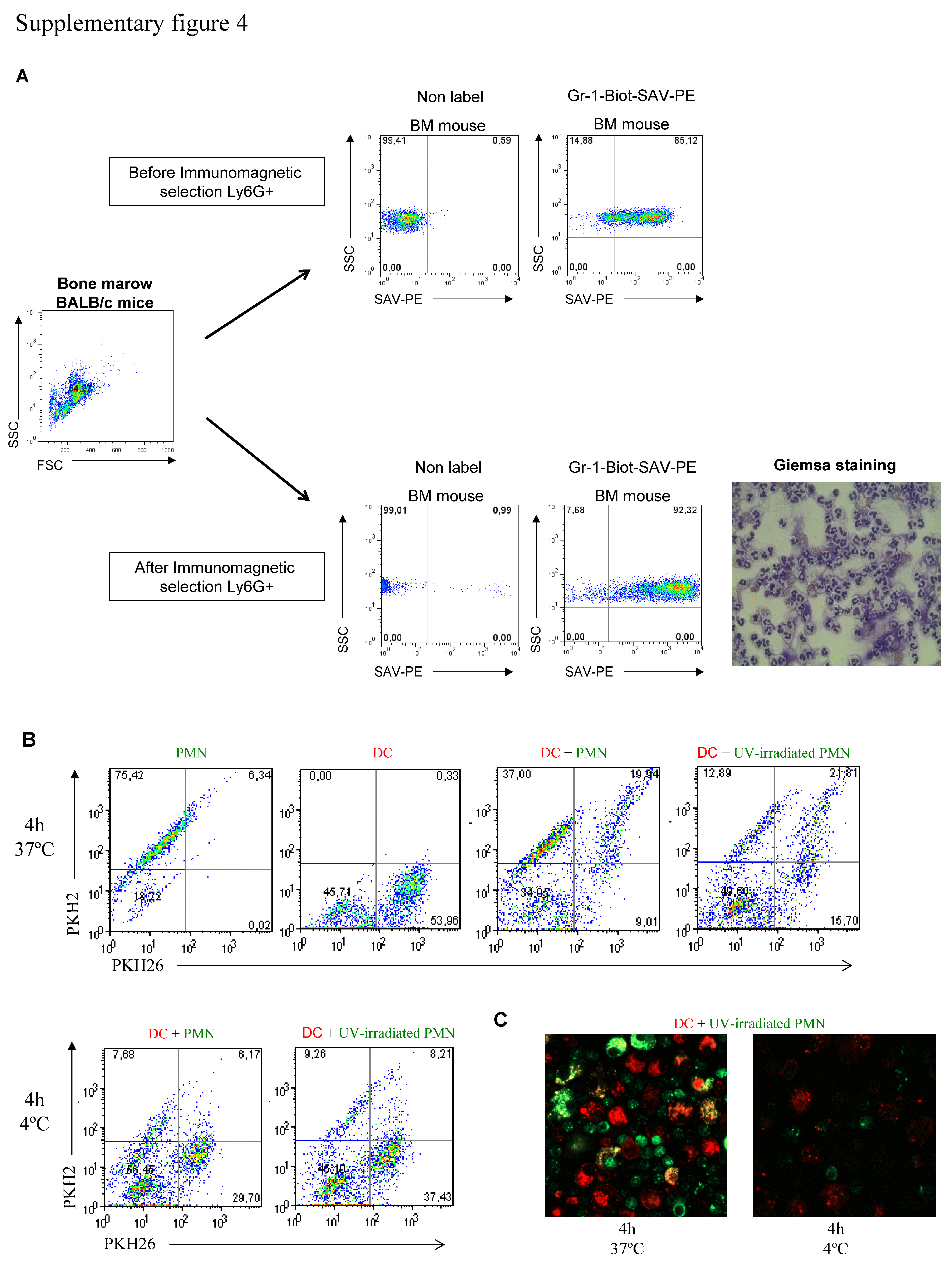

Supplement: Figure S4 — Purification of mouse neutrophils and co-cultures with mouse DC. (A) Immunomagnetic selection of Ly-6G cells from single cell suspensions from mouse femur and tibia bone-marrow before and after immunomagnetic selection (Automacs®). Purity was assessed by cells showing bright Gr-1 immunostaining and upon May-Grünwald Giemsa stainings of cytospins. (B) FACS dot plot analysis of mouse DC and PMN labeled with PKH2 and PKH26 as a single cell type or in co-culture as indicated. 4 h co-cultures were performed at 37°C or 4°C as indicated. (C) Representative confocal images of co-cultures set up with the PMN and DC at the indicated temperature conditions. (TIF) [file pone.0029300.s004.tif]

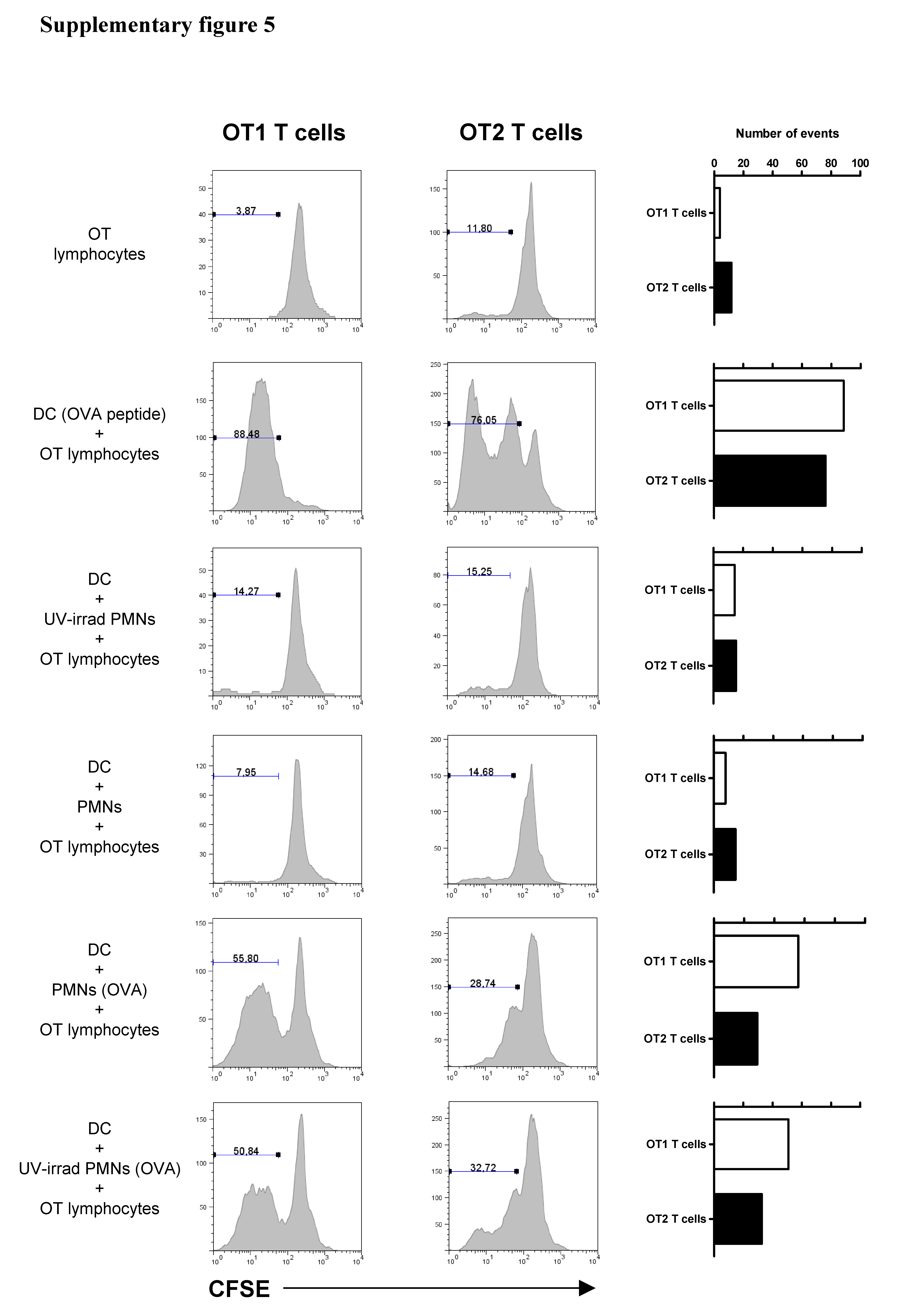

Supplement: Figure S5 — Individual histograms from the overlay shown in figure 7B. Histograms and graphs indicate dilution of CFSE that was triggered by the cognate peptides pulsed on DC as a positive control and by DC that had been incubated with PMNs pre-loaded or not with OVA. (TIF) [file pone.0029300.s005.tif]

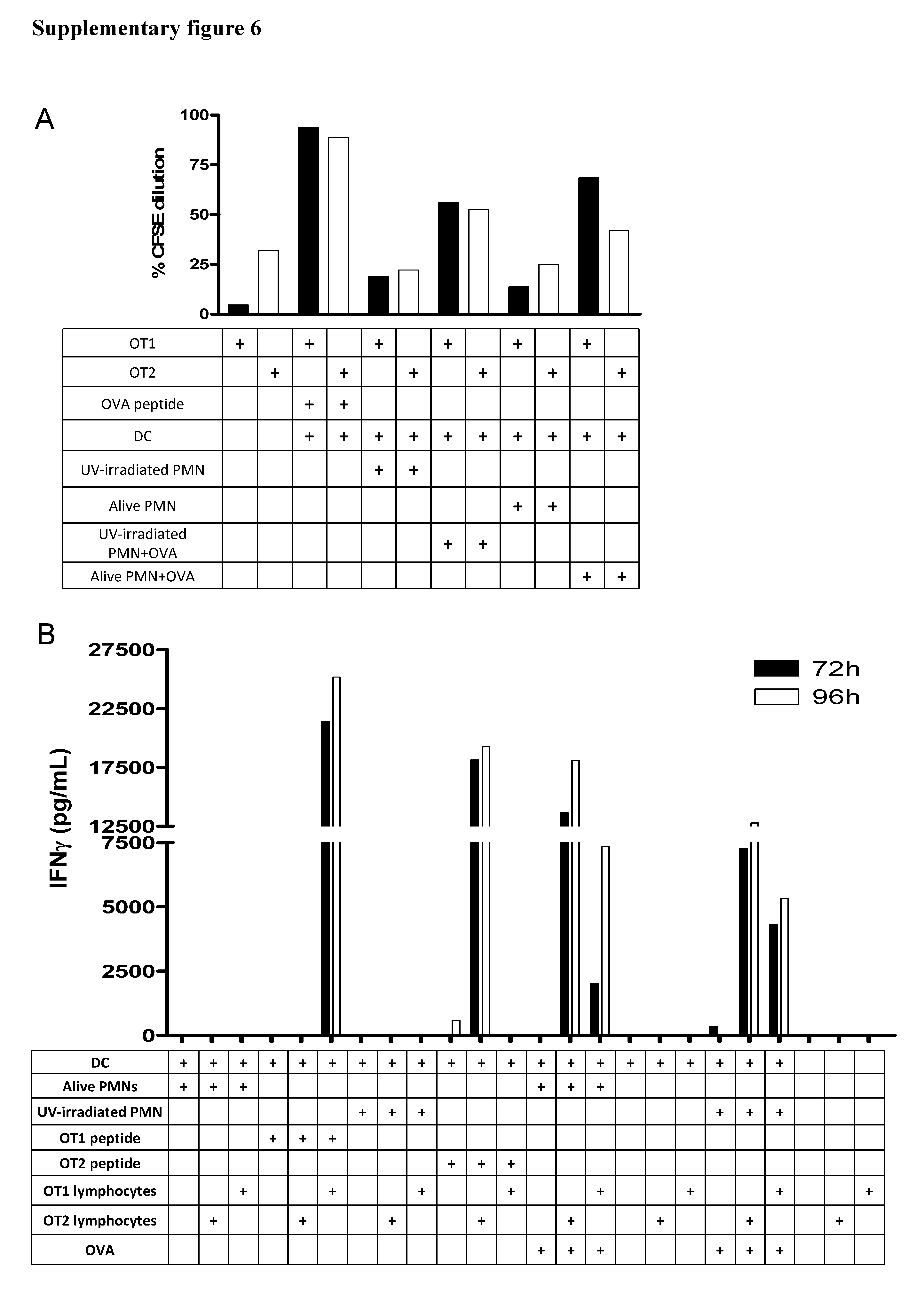

Supplement: Figure S6 — CFSE dilution and IFNγ concentrations of OT-1 and OT-2 cell cultures (A) Shows the extent of CFSE dilution in OT-1 and OT-2 cells. In a replicate experiment performed identically to the one shown in figure 7B. (B) IFNγ concentrations released in 72 h and 96 h to the supernatant of cultures by OT-1 and OT -2 lymphocytes in conditions identical to those in figure 7 (as indicated in the figure) in the same experiment in which CFSE dilution was monitored in A. (TIF) [file pone.0029300.s006.tif]

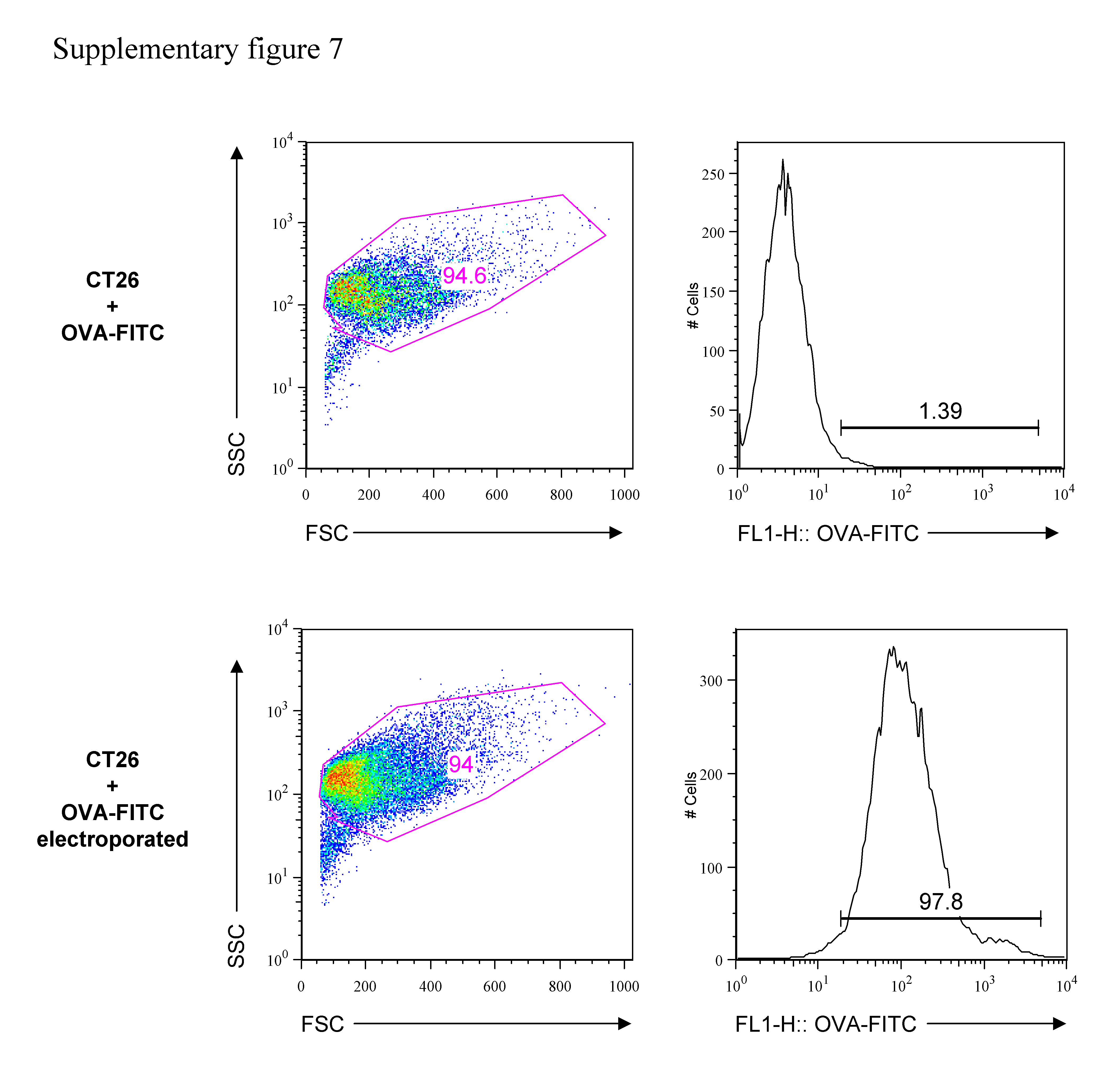

Supplement: Figure S7 — Dot plots FSC/SSC and green fluorescence (FL1) of CT26 tumor cells set in the presence of OVA-FITC and electroporated or mock-electroporated as indicated. Cells were analyzed by flow cytometry for FITC fluorescence 2 h following electroporation or mock electroporation. (TIF) [file pone.0029300.s007.tif]
